# Invited Commentary: Overall Survival in Patients with Stage IV Pan-NET Eligible for Liver Transplantation

**DOI:** 10.1007/s00268-022-06749-w

**Published:** 2022-10-11

**Authors:** Inne H. M. Borel Rinkes

**Affiliations:** Department of Endocrine Surgery, GI Surgery and Surgical Oncology, UMC-Utrecht Cancer Center, University Medical Center Utrecht, Utrecht, The Netherlands

The treatment of hepatic metastases from neuro-endocrine tumors (NET) remains complex and challenging, while research on this topic is greatly hampered by the low incidence and a high degree of heterogeneity, both between patients and reported studies. This is particularly true for liver metastases originating from pancreatic NETs (pNET). Hepatic metastatic pNET is often considered unresectable due to the diffuse metastatic pattern that is generally observed. Alternative therapeutic options, including locally ablative techniques, PRRT, transarterial radio- or chemo-embolization, chemotherapy and molecular targeting agents, are all being used in multimodal approaches, while liver transplantation (LTx) has been advocated in patients with liver-only disease in whom the primary NET has been completely resected. According to a relatively recent systematic review, 5-year survival after LTx for metastatic NETs of pancreatic and gastro-intestinal origin together appears to lie around 60–65% [1]. Pancreatic location of the primary NET was found to be associated with worse outcome when compared with gastro-intestinal NETs.

In their very interesting article in this edition of WJS, Kjaer and co-authors challenge the indication for LTx in metastatic pNET [2]. In this retrospective mono-institutional study on 41 patients who might be considered for transplantation based on reasonable age (< 75 years), favorable tumor biology (grade ≤ 2) and absence of extra-abdominal metastases, but were treated with multimodal medical therapy, they found an overall 5-year survival of 64.7%. In patients meeting either one of three existing internationally accepted criteria guidelines for LTx in mNET (*n* = 16), 5-year survival ranged from 55.4 to 85.7%. For the purpose of comparison, the authors extracted those patients with true *pancreatic* NETs from published literature on LTx for metastatic NET (where published data sets often include a mix of pancreatic and gastro-intestinal NETs) and found those to be inferior (varying between 27 and 44%). Hence, the authors propose that the indication for LTx in such patients is based on weak evidence and should be reconsidered, particularly when taking the morbidity and mortality associated with LTx into account. They advocate to embark on a randomized trial, comparing LTx with multimodal medical treatment, admitting that this would be very hard to accomplish.

In contrast to these findings, Mazzaferro et al. reported a significant survival benefit of LTx in patients with hepatic metastases from NETs [3]. In that study, 88 consecutive patients with hepatic metastases from NETs, eligible for LTx according to the Milan-NET criteria, were offered LTx (*n* = 42) versus non-transplant (*n* = 46) treatment, based on patient compliance, list dynamics and age considerations. Bias was reduced by means of propensity score matching, resulting in similar hepatic tumor burden and % of pancreatic NET primary tumors between groups. The respective 5- and 10-year survival rates were 97.2 and 88.8% for the LTx group versus 50.9 and 22.4% for non-LTx patients. The survival advantage of the LTx group was significant with a hazard ratio of 7.4. Although Kjaer et al. argue—correctly—that patients in the LTx group were younger and had lower TNM stage and tumor grade of their primary NETs, they all had substantial and comparable metastatic liver disease, and the reported survival differences are enormous. However, Kjaer et al. raise another interesting point, i.e. that immortal time bias could have been substantial in the Mazzaferro study where survival was measured from surgery of the primary and not from transplantation (-eligibility) date. They have corrected for this in their current report where *t* = 0 was defined as the moment that patients were actually designated eligible for LTx. This is indeed an important issue that should be taken into account in any future study regarding outcome in metastatic pNET.

Kjaer et al. are to be commended for their attempts to extract data on LTx for metastasized pancreatic NET from literature reports, the majority of which have grouped together all varieties of GEP-NET primary tumors together in their analyses. In addition, they are correct in stating that LTx is associated with significant morbidity and even mortality. Nonetheless, it would have been helpful if they had provided data concerning unwanted side effects resulting from multimodal medical treatment scenario’s, as these will all have to be considered and communicated with our patients in this difficult category.

While the Mazzaferro data cannot be ignored, the Kjaer report is sufficiently provocative to pursue. Although theoretically and statistically optimal, a randomized trial addressing this rare condition is simply not realistic. Hence, I would propose to assemble a much larger, multi-institutional data set with a fixed *t* = 0 on multimodal treatment for metastatic pNET in an attempt to corroborate the results put forward by Kjaer et al. Until then, we must continue to weigh the reported survival data in literature against the associated morbidity and mortality of LTx versus multimodal treatment for each individual patient with pancreatic NET metastasized to the liver meeting the current transplant criteria.
